# Association of Haptoglobin Phenotype With Neurological and Cognitive Outcomes in Patients With Subarachnoid Hemorrhage

**DOI:** 10.3389/fnagi.2022.819628

**Published:** 2022-03-21

**Authors:** Sung Woo Han, Bong Jun Kim, Tae Yeon Kim, Seung Hyuk Lim, Dong Hyuk Youn, Eun Pyo Hong, Jong Kook Rhim, Jeong Jin Park, Jae Jun Lee, Yong Jun Cho, Ben Gaastra, Ian Galea, Jin Pyeong Jeon

**Affiliations:** ^1^Institute of New Frontier Research, Hallym University College of Medicine, Chuncheon, South Korea; ^2^Department of Neurosurgery, Jeju National University School of Medicine, Jeju, South Korea; ^3^Department of Neurology, Konkuk University Medical Center, Seoul, South Korea; ^4^Department of Neurosurgery, Hallym University College of Medicine, Chuncheon, South Korea; ^5^Department of Neurosurgery, Wessex Neurological Centre, University Hospital Southampton, Southampton, United Kingdom; ^6^Clinical Neurosciences, Clinical and Experimental Sciences, Faculty of Medicine, University of Southampton, Southampton, United Kingdom

**Keywords:** subarachnoid hemorrhage, haptoglobin, cognition, outcome, intracranial aneurysm

## Abstract

**Background:**

To assess the association of haptoglobin (Hp) phenotype with neurological and cognitive outcomes in a large cohort of patients with subarachnoid hemorrhage (SAH).

**Methods:**

This prospective multicenter study enrolled patients with aneurysmal SAH between May 2015 and September 2020. The Hp phenotype was confirmed *via* Western blots. The relative intensities of α1 in individuals carrying Hp2-1 were compared with those of albumin. Multivariable logistic and Cox proportional-hazard regression analyses were used to identify the risk factors for 6-month and long-term outcomes, respectively.

**Results:**

A total of 336 patients including the phenotypes Hp1-1 (*n* = 31, 9.2%), Hp2-1 (*n* = 126, 37.5%), and Hp2-2 (*n* = 179, 53.3%) were analyzed. The Hp phenotype was closely associated with 6-month outcome (*p* = 0.001) and cognitive function (*p* = 0.013), and long-term outcome (*p* = 0.002) and cognitive function (*p* < 0.001). Compared with Hp1-1 as the reference value, Hp2-2 significantly increased the risk of 6-month poor outcome (OR: 7.868, 95% CI: 1.764–35.093) and cognitive impairment (OR: 8.056, 95% CI: 1.020–63.616), and long-term poor outcome (HR: 5.802, 95% CI: 1.795–18.754) and cognitive impairment (HR: 7.434, 95% CI: 2.264–24.409). Long-term cognitive impairment based on the Hp phenotype was significantly higher in patients under 65 years of age (*p* < 0.001) and female gender (*p* < 0.001). A lower relative α1/albumin intensity (OR: 0.010, 95% CI: 0.000–0.522) was associated with poor outcome at 6 months but not cognitive impairment in patients with SAH expressing Hp2-1.

**Conclusion:**

Hp2-2 increased the risk of poor neurological outcomes and cognitive impairment compared with Hp1-1. For Hp2-1, higher relative α1 intensities were related to 6-month favorable outcomes.

## Introduction

Aneurysmal subarachnoid hemorrhage (SAH) triggers acute hemorrhage into the subarachnoid space that contains cerebrospinal fluid (CSF) and surrounds the cerebral vasculature. SAH leads to increased intracranial pressure and decreased cerebral blood flow in the brain, in particular, during the early phase after aneurysm rupture (Fujii et al., 2013). Inflammatory response and oxidative stress triggered by free hemoglobin (Hb) following hemolysis within the CSF space causes vasospasm, and in severe cases, neurological deficits (Froehler et al., 2010). Thus, even if the intracranial aneurysm is secured, these changes may exacerbate inflammation and oxidative injury, and disruption of the blood-brain barrier (BBB) (Fujii et al., 2013), which ultimately lead to poor physical and cognitive function.

Within the CSF space, haptoglobin (Hp) binds to free Hb and is then eliminated by macrophages *via* the cell surface receptor CD163 (Galea et al., 2012; Gaastra et al., 2017). Hp consists of two light alpha (α) and two heavy (β) chains. In humans, there are three types of Hp: Hp1-1 (two α1 chains), Hp2-1 (α1 and α2 chains), and Hp2-2 (two α2 chains) (Gaastra et al., 2017; Kim et al., 2018). The Hp-Hb complex is rapidly cleared compared with free Hp (Gueye et al., 2006), but the effects of clearance vary according to the Hp type. It was generally believed that patients with SAH with Hp1-1 manifested better outcomes and a lower incidence of delayed cerebral ischemia (DCI) compared with those with Hp2-2 (Bamm et al., 2004; Gaastra et al., 2017). However, most of the studies were based on a sample size of less than 100 patients with SAH, and the association of the Hp phenotype with neurological outcomes differed across studies (Ohnishi et al., 2013; Leclerc et al., 2015; Kim et al., 2018). Kim et al. (2018) reported that patients with SAH expressing Hp2-2 had significantly higher rates of DCI than those carrying Hp1-1 (53.8% in Hp2-2 vs. 25.0% in Hp1-1). Leclerc et al. (2015) also showed that the Hp2-2 phenotype is an independent risk factor for poor functional outcomes and mortality. Meanwhile, Ohnishi et al. (2013) reported that Hp2-2 was not associated with 3-month functional outcomes, although it significantly increased the risk of angiographic vasospasm. In addition, the Hp2 allele has been associated with long-term favorable neurologic outcomes in patients with SAH at a high Fisher grade (Morton et al., 2020). Therefore, the association between the Hp phenotype and outcomes has yet to be demonstrated in a large cohort of patients with SAH.

In this study, we focused on the following three issues. First, the association between the Hp phenotype and the long-term outcomes remains undetermined. Most of the studies were limited to outcomes within 6 months after ictus and only included a small number of patients. Second, previous studies have focused on cerebral vasospasm, and DCI, and not on the association of the Hp phenotype with cognitive impairment. Cognitive impairment does not necessarily correlate with neurological outcomes and consequently should be analyzed separately (Wong et al., 2014). In addition, even in patients who have recovered well, cognitive impairment is frequently observed (Buunk et al., 2019). Third, the outcomes in patients with SAH expressing Hp2-1 are seldom reported, although differences in the protective effect of Hp2-2 and Hp1-1 have been widely reported. To address the three issues, we have analyzed the associations between the Hp phenotype and neurological and cognitive outcomes in a large cohort of patients with SAH *via* long-term follow-up. In addition, we measured the relative α1/albumin intensity in individuals with Hp2-1 (Kim et al., 2018) and investigated its role as a potential biomarker of outcomes in patients with SAH expressing Hp2-1.

## Materials and Methods

### Participants

The SAH cohort was obtained from “The First Korean Stroke Genetics Association Research” study, which prospectively collected data of patients with various cerebrovascular diseases such as SAH, intracranial hemorrhage, arteriovenous malformation, and moyamoya disease at five university hospitals since 2015 (Kim et al., 2020; Park et al., 2020). This prospective multicenter study involved a Biobank of patient samples such as blood, CSF, and tissues as well as patients’ clinical and radiological data. This database was used to select patients with SAH recruited prior to September 2020 based on the following criteria: (1) adult patients with SAH aged ≥18 years, (2) spontaneous SAH due to saccular aneurysm rupture, and (3) Hp phenotype identified *via* Western blot. The exclusion criteria were (1) non-aneurysmal SAH due to trauma or infection, (2) angiogram-negative SAH, (3) SAH accompanied by other cerebrovascular diseases such as arteriovenous malformation or moyamoya disease, (4) follow-up loss, and (5) insufficient medical data to determine patient’s outcome (Kim et al., 2020). In the case of SAH, Western blotting was performed with blood obtained within 24 h after patient’s hospitalization to determine the association with various outcomes according to the Hp phenotypes. When examining cognitive impairment in SAH survivors, additional items were incorporated under the existing inclusion and exclusion criteria, respectively. The additional inclusion criteria were Mini-Mental State Examination (MMSE) conducted periodically and communication skills. Additional exclusion criteria were inability to communicate and death and dementia diagnosed at the time of admission, or cases of refusal to undergo MMSE test.

Treatment protocols of the five hospitals enrolled in this study were as follows. All patients with SAH who visited the emergency department underwent cerebral angiography immediately after diagnosis, followed by prompt endovascular coil embolization or surgical clipping. In general, continuous lumbar drainage was maintained until 7 days after the treatment. Nimodipine (Samjin Pharmaceutical, Seoul, South Korea) was administered intravenously at a dose of 20 μg/kg/h to prevent cerebral vasospasm (Park et al., 2021).

### Study Outcomes

The primary outcome was the association between the Hp phenotype and neurological outcome at 6 months and long-term follow-up. Secondary outcomes were cognitive impairment according to the Hp phenotype at 6 months and long-term follow-up. We further analyzed outcomes in patients with SAH carrying Hp2-1 based on the relative intensity of α1 expression (Kim et al., 2018). Poor neurological outcome was defined as a modified Rankin Scale (mRS) score of 3–6. Cognitive performance was assessed using the Korean version of the MMSE (K-MMSE). The assessment was routinely performed at 6 months following SAH, and thereafter, periodically at 1-year intervals. An MMSE score of less than 27 indicates cognitive impairment (King et al., 2006; Kim et al., 2012; Gluhm et al., 2013). The medical records including clinical variables (e.g., gender, age, hypertension, diabetes mellitus, hyperlipidemia, and smoking), radiological variables (e.g., Hunt and Hess (H-H) grade, Fisher grade, hydrocephalus, and vasospasm), and treatment methods were reviewed (Jeon et al., 2012). The H-H grade was divided into two groups: lower H-H (I, II, and III) and higher H-H group (IV and V), and likewise Fisher grade was divided into a low grade (Fisher grades I and II) and high grade (Fisher grades III and IV) (Kim et al., 2018). Severe cerebral vasospasm was defined as a decrease in vessel diameter of over 70% compared with baseline computed tomography angiography or magnetic resonance angiography (Park et al., 2021). This study was approved by the Institutional Review Boards (IRB No: 2016-31, 2018-6, and 2019-06). Informed consent was obtained from patients or their legal representatives.

### Haptoglobin Phenotyping

The blood samples of all patients with SAH were obtained within 24 h after admission, followed by Hp phenotyping. Blood collected from the peripheral artery or vein was centrifuged at 4,000 rpm for 5 min. The serum was collected and stored at −80°C before analysis. The Hp phenotyping was performed *via* polyacrylamide gel electrophoresis, followed by immunoblotting to detect differences in α1 and α2 chain size according to previously reported protocols (Leclerc et al., 2015; Kim et al., 2018). First, the Hp type was determined, followed by Western blotting analysis in Hp2-1 individuals using a serum diluted in a 1:75 ratio obtained by adding 1 μl of serum to 74 μl of phosphate-buffered saline. Samples were prepared by mixing the serum diluent with an equal volume of 2× SDS sample buffer (Bio-Rad, Hercules, CA, United States) and boiled at 95°C for 8 min. Next, a 10 μl aliquot of the sample was loaded on 15% of polyacrylamide gel and electrophoresed for 150 min at 100 V (Bio-Rad, Hercules, CA, United States), followed by transfer to the membranes, which were then blocked with 5% of BSA in TBST (10 mM Tris–HCl pH8.0, 150 mM NaCl) including 0.01% Tween-20 for 1 h. The membranes were incubated overnight with polyclonal rabbit antihuman haptoglobin antibody (Dako, Glostrup, Denmark) diluted in a 1:10,000 ratio in blocking buffer at 4°C. After washing three times with TBST, the membranes were incubated with horseradish peroxidase (HRP)-conjugated goat anti-rabbit IgG (Abcam, Cambridge, United Kingdom) in a 1:10,000 ratio for 1 h at room temperature. Following a final washing step, the membranes were treated with HRP substrate (Thermo Fisher Scientific, Waltham, MA, United States), and chemiluminescence was detected using an X-ray film (Kodak, Rochester, NY, United States). Additional immunoprecipitation was carried out with an anti-Hp antibody to determine the polymeric composition of Hp2-1 based on the molecular size. To immunoprecipitate Hp, 0.3 mg/ml of the serum obtained from a patient with Hp2-1 was incubated overnight with an anti-Hp antibody at 4°C. The immune complexes were precipitated with protein A/G Sepharose (Santa Cruz, Dallas, TX, United States), followed by Western blotting. Anti-albumin antibody (Abcam, Cambridge, United Kingdom) was used as a loading control. The relative α1 intensity was obtained by dividing the α1 intensity value by albumin intensity. The band intensities of α1 chains and albumin were analyzed using ImageJ software (version 1.49, National Institutes of Health, Bethesda, MD, United States) (Kim et al., 2018).

### Statistical Analysis

Discrete data are presented as means, while proportions and continuous data are expressed as means ± standard deviation (SD). Statistical methods such as Chi-squared or Student’s *t*-test were used as appropriate. Univariate analysis was performed to identify relevant factors associated with outcomes. Multivariable logistic regression analysis was performed to further identify independent variables associated with 6-month neurological and cognitive outcomes. Long-term outcomes were assessed using Cox proportional-hazards regression. Cumulative survival plots of the cognitive impairment-free patients based on the Hp phenotype were generated using the Kaplan-Meier product estimator (Jeon et al., 2014). Additionally, their association was further analyzed based on age (<65 years vs. ≥65 years) and gender (male vs. female). The relative intensities of Hp2-1 individuals were calculated by dividing the α1 intensity value by the albumin intensity and correlated with outcomes based on a previous report (Kim et al., 2018). The *p-*value < 0.05 was regarded as statistically significant. Statistical analyses were conducted using SPSS version 19 (SPSS, Armonk, NY, United States) and MedCalc (www.MedCalc.org accessed on March 1, 2021).

## Results

### Baseline Characteristics

**FIGURE 1:**
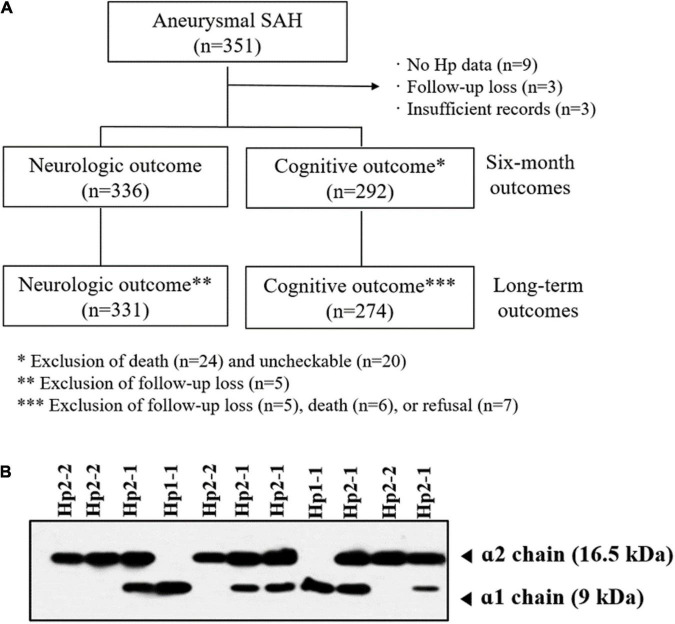
**(A)** Flow chart outlining the study protocol. **(B)** Western blotting analysis of haptoglobin (Hp) phenotype.

A total of 351 patients were initially enrolled. After excluding patients with insufficient medical records, absence of Hp phenotype, and follow-up loss, 336 patients were included in the analysis (Figure 1A). The numbers of patients according to the Hp phenotypes were as follows: Hp1-1, *n* = 31 (9.2%); Hp2-1, *n* = 126 (37.5%); and Hp2-2, *n* = 179 (53.3%) (Figure 1B). The mean age according to the Hp phenotypes did not differ significantly: Hp1-1, 58.7 ± 13.9 years; Hp2-1, 62.5 ± 12.4 years; and Hp2-2, 60.0 ± 11.5 years. Coil embolization and surgical clipping were performed in 256 (76.2%) and 80 (23.8%) patients, respectively. Most aneurysms were located in the anterior circulation. Six-month poor outcomes and cognitive impairments were more frequently observed in patients with Hp2-2 compared with Hp1-1 and Hp2-1. Except for these two variables, there was no significant difference in the variables according to the Hp phenotype. Detailed information is presented in Table 1.

**TABLE 1:** Demographic and clinical characteristics of patients with subarachnoid hemorrhage according to haptoglobin (Hp) phenotype.

	Hp phenotypes			
Variables	Hp1-1 (*n* = 31)	Hp2-1 (*n* = 126)	Hp2-2 (*n* = 179)	*p*-value
**Clinical variables**				
Female	20 (64.5%)	86 (68.3%)	120 (67.0%)	0.920
Age (years)	58.7 ± 13.9	62.5 ± 12.4	60.0 ± 11.5	0.122
Hypertension	11 (35.5%)	68 (54.0%)	88 (49.2%)	0.179
Diabetes mellitus	5 (16.1%)	18 (14.3%)	19 (10.6%)	0.516
Hyperlipidemia	4 (12.9%)	22 (17.5%)	27 (15.1%)	0.769
Smoking	6 (19.4%)	13 (10.3%)	20 (11.2%)	0.359
**Radiological variables**				
Hunt and Hess grade IV and V	12 (38.7%)	57 (45.2%)	91 (50.8%)	0.365
Fisher grade III and IV	18 (58.1%)	64 (50.8%)	101 (56.4%)	0.570
Anterior circulation aneurysm	27 (87.1%)	107 (84.9%)	156 (87.2%)	0.848
Hydrocephalus	8 (25.8%)	12 (9.5%)	24 (13.4%)	0.054
Severe vasospasm	10 (32.3%)	32 (25.4%)	47 (26.3%)	0.736
**Treatment method**				
Coil embolization	23 (74.2%)	95 (75.4%)	138 (77.1%)	0.908
**Outcomes**				
Poor outcome on 6 months	2 (6.5%)	26 (20.6%)	63 (35.2%)	0.001
Cognitive impairment on 6 months*	1 (3.3%)	11 (9.7%)	30 (20.1%)	0.011

*Six-month cognitive impairment was analyzed in 292 patients including 30 patients with Hp1-1, 113 with Hp2-1, and 149 with Hp2-2.

### Neurological Outcomes

A total of 336 patients were analyzed to assess the relationship between the Hp phenotypes and poor outcomes at 6 months. Poor outcome was observed in 91 patients (27.1%). Multivariate analysis revealed that advanced age (OR: 1.031, 95% CI: 1.007–1.055), high H-H (OR: 2.823, 95% CI: 1.576–5.055) and Fisher grades (OR: 2.185, 95% CI: 1.198–3.984), and Hp phenotypes were closely associated with poor outcomes. When Hp1-1 was used as a reference value, Hp2-2 significantly increased the risk of poor outcomes (OR: 7.868, 95% CI: 1.764–35.093). The long-term outcome was analyzed in 331 patients with a mean follow-up of 34.6 ± 13.7 months. It was linked to advanced age (HR: 1.024, 95% CI: 1.005–1.042), high H-H (HR: 2.047, 95% CI: 1.337–3.134), hydrocephalus (HR: 1.754, 95% CI: 1.057–2.909), and Hp phenotype. With Hp1-1 as the reference value, the presence of Hp2-2 (HR: 5.802, 95% CI: 1.795–18.754) and Hp2-1 (HR: 3.522, 95% CI: 1.049–11.828) significantly increased the risk of long-term poor outcomes following SAH (Table 2).

**TABLE 2:** Multivariable logistic regression analysis of factors associated with 6-month poor outcomes and Cox proportional-hazards regression analysis of long-term poor outcomes following subarachnoid hemorrhage.

	6-month poor outcomes*		Long-term poor outcomes**	
Variables	OR (95% CI)	*p*-value	HR (95% CI)	*p*-value
Clinical variables				
Female	0.877 (0.476−1.617)	0.674	0.769 (0.503−1.177)	0.227
Age, years	1.031 (1.007−1.055)	0.011	1.024 (1.005−1.042)	0.012
Hypertension	1.037 (0.582−1.847)	0.903	1.139 (0.739−1.756)	0.556
Diabetes mellitus	1.201 (0.548−2.632)	0.646	1.401 (0.787−2.494)	0.252
Hyperlipidemia	0.564 (0.253−1.255)	0.160	0.804 (0.412−1.568)	0.522
Smoking	0.859 (0.370−1.997)	0.724	1.079 (0.580−2.006)	0.810
Radiological variables				
Hunt and Hess grade IV and V	2.823 (1.576−5.055)	<0.001	2.047 (1.337−3.134)	0.001
Fisher grade III and IV	2.185 (1.198−3.984)	0.011	1.488 (0.907−2.443)	0.116
Anterior circulation aneurysm	1.116 (0.528−2.359)	0.774	1.570 (0.922−2.674)	0.097
Hydrocephalus	1.468 (0.684−3.152)	0.324	1.754 (1.057−2.909)	0.030
Severe vasospasm	1.425 (0.794−2.558)	0.235	1.302 (0.846−2.003)	0.230
Treatment methods				
Coil embolization	0.925 (0.491−1.742)	0.809	0.945 (0.593−1.507)	0.812
Hp phenotypes		0.001		0.002
Hp1-1	1 (reference)		1 (reference)	
Hp2-1	3.406 (0.737-15.746)	0.117	3.522 (1.049-11.828)	0.042
Hp2-2	7.868 (1.764–35.093)	0.007	5.802 (1.795–18.754)	0.003

*, ** Six-month outcomes were analyzed in 336 patients and the long-term outcomes involved 331 patients. OR, odds ratio; CI, confidence interval; HR, hazards ratio.

### Cognitive Impairment

The 6-month cognitive outcome was analyzed in 292 patients diagnosed with SAH (Figure 1A). The rates of cognitive impairment based on Hp phenotype were as follows: Hp1-1, 3.3% (1 out of 30); Hp2-1, 9.7% (11 out of 113); and Hp2-2, 20.1% (30 out of 149). Cognitive impairment was strongly related to age (OR: 1.034, 95% CI: 1.001–1.068), high H-H (OR: 2.547, 95% CI: 1.162–5.583) and Fisher grades (OR: 3.359, 95% CI: 1.409–8.012), and Hp phenotype. Hp2-2 phenotype significantly increased the risk of 6-month cognitive impairment compared with Hp1-1 (OR: 8.056, 95% CI: 1.020–63.616) (Table 3). Long-term cognitive outcome was analyzed in 274 patients with a mean follow-up of 32.3 ± 16.7 months. The distribution of cognitive impairment based on Hp phenotype was as follows: Hp1-1, *n* = 3 (10.3%); Hp2-1, *n* = 18 (17.6%); and Hp2-2, *n* = 61 (42.7%). Risk factors associated with long-term cognitive impairment were advanced age (HR: 1.033, 95% CI: 1.012–1.055), high H-H grade (HR: 2.598, 95% CI: 1.616–4.177), hydrocephalus (HR: 2.048, 95% CI: 1.154–3.633), and Hp phenotype. Compared with Hp1-1, Hp2-2 was associated with significant long-term cognitive impairment (HR: 7.434, 95% CI: 2.264–24.409).

**TABLE 3:** Multivariable logistic regression analysis of variables associated with 6-month cognitive impairment and Cox proportional-hazards regression analysis of long-term cognitive impairment following subarachnoid hemorrhage.

	6-month cognitive impairment*		Long-term cognitive impairment**	
Variables	OR (95% CI)	*p*-value	HR (95% CI)	*p*-value
Clinical variables				
Female	0.878 (0.372−2.074)	0.768	0.801 (0.495−1.296)	0.366
Age	1.034 (1.001−1.068)	0.043	1.033 (1.012−1.055)	0.002
Hypertension	0.631 (0.296−1.344)	0.233	1.104 (0.699−1.746)	0.671
Diabetes mellitus	0.987 (0.311−3.127)	0.982	1.181 (0.603−2.314)	0.628
Hyperlipidemia	0.366 (0.101−1.325)	0.126	0.893 (0.468−1.707)	0.733
Smoking	0.841 (0.254−2.781)	0.776	1.034 (0.527−2.028)	0.923
Radiological variables				
Hunt and Hess grade IV and V	2.547 (1.162−5.583)	0.020	2.598 (1.616−4.177)	<0.001
Fisher grade III and IV	3.359 (1.409−8.012)	0.006	1.460 (0.867−2.460)	0.155
Anterior circulation aneurysm	0.470 (0.169−1.309)	0.148	0.932 (0.410−2.115)	0.866
Hydrocephalus	1.901 (0.677−5.335)	0.223	2.048 (1.154−3.633)	0.014
Severe vasospasm	1.021 (0.464−2.247)	0.959	1.249 (0.777−2.010)	0.358
Treatment methods				
Coil embolization	1.088 (0.443−2.672)	0.854	0.863 (0.498−1.496)	0.601
Hp phenotypes		0.013		<0.001
Hp1-1	1 (reference)		1 (reference)	
Hp2-1	3.057 (0.365−25.620)	0.303	2.854 (0.815−9.997)	0.101
Hp2-2	8.056 (1.020−63.616)	0.048	7.434 (2.264−24.409)	0.001

*, ** Six-month cognitive impairment was analyzed in 292 patients and long-term cognitive impairment was assessed in 274 patients. OR, odds ratio; CI, confidence interval; HR, hazards ratio.

**FIGURE 2:**
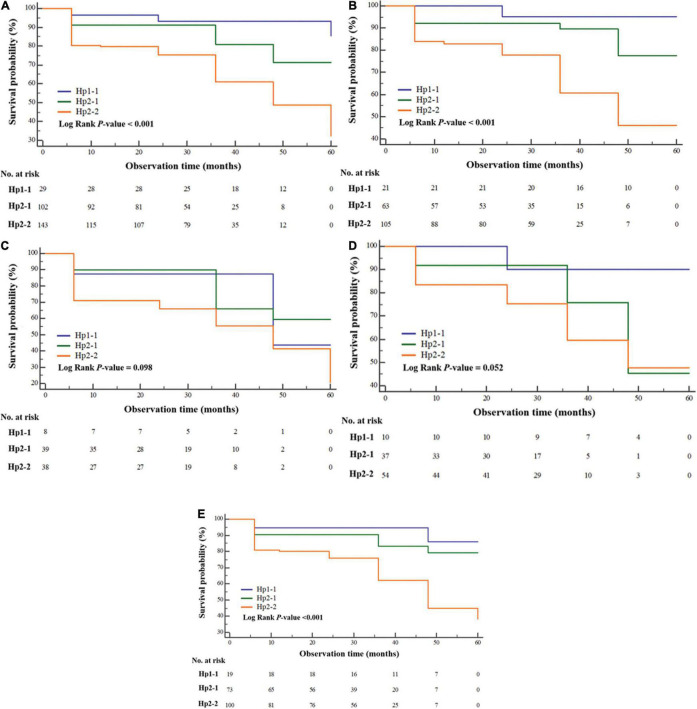
Kaplan-Meier survival curve illustrating the cumulative cognitive impairment-free survival probability according to haptoglobin phenotype **(A)** and subgroup analyses of patients aged <65 years **(B)** and ≥65 years, including **(C)** men **(D)** and women **(E)** after subarachnoid hemorrhage.

Cumulative survival plots of impairment-free cognition according to Hp phenotype revealed that patients with SAH expressing Hp1-1 (HR: 0.159, 95% CI: 0.084–0.299) and Hp2-1 (HR: 0.416, 95% CI: 0.258–0.671) had less cognitive impairment compared with those manifesting Hp2-2 (*p* for log-rank test < 0.001) (Figure 2A). We further analyzed long-term cognitive impairment after adjusting for age (Figures 2B,C) and gender (Figures 2D,E). The associations were clearly evident in patients younger than 65 years of age (*p* < 0.001) and in women (*p* < 0.001) compared with those aged above 65 years (*p* = 0.098) and male gender (*p* = 0.052).

### Analysis of Outcomes in Patients With Hp2-1

In case of patients diagnosed with SAH expressing Hp2-1 phenotype alone, the risk factors for outcomes were further analyzed (*n* = 126) (Table 4). Poor outcome at 6 months was observed in 26 patients (20.6%). The relative α1/albumin intensities in individuals with favorable and poor outcomes were 0.31 ± 0.13 and 0.22 ± 0.37, respectively (*p* = 0.041). Multivariate analysis revealed that high H-H grade (OR: 4.500, 95% CI: 1.690–11.983) and relative α1 intensity (OR: 0.010, 95% CI: 0.000–0.522) were associated with poor outcomes. High Fisher grade was a significant risk factor for 6-month (OR: 11.702, 95% CI: 1.444–94.816) and long-term cognitive impairment (HR: 8.140, 95% CI: 1.021–64.880). Relative α1 intensity was not significantly associated with 6-month (OR: 2.589, 95% CI: 0.116–57.989) or long-term cognitive impairment (HR: 3.813, 95% CI: 0.312–46.644).

**TABLE 4:** Multivariable logistic regression analysis of variables associated with poor neurological outcome and cognitive impairment at 6 months, and Cox proportional-hazards regression analysis of long-term cognitive impairment during follow-up of patients with subarachnoid hemorrhage expressing haptoglobin 2-1.

	6-month poor outcome		6-month cognitive impairment		Long-term cognitive impairment	
	(*n* = 126)		(*n* = 113)*		(*n* = 102)**	
Variables	OR (95% CI)	*p*-value	OR (95% CI)	*p*-value	HR (95% CI)	*p*-value
**Clinical variables**						
Female	1.053 (0.309−3.588)	0.935	1.156 (0.201−6.633)	0.871	1.059 (0.152−7.369)	0.954
Age, years	1.021 (0.981−1.062)	0.311	1.018 (0.957−1.082)	0.579	1.017 (0.961−1.075)	0.558
Hypertension	1.508 (0.529−4.299)	0.442	0.451 (0.119−1.716)	0.243	0.879 (0.193−3.998)	0.867
Diabetes mellitus	0.923 (0.210−4.061)	0.915	0.512 (0.045−5.858)	0.591	0.461 (0.050−4.288)	0.496
Hyperlipidemia	0.644 (0.170−2.440)	0.517	0.270 (0.022−3.384)	0.310	0.377 (0.046−3.065)	0.362
Smoking	0.283 (0.033−2.429)	0.250	0.361 (0.037−3.561)	0.383	0.310 (0.026−3.687)	0.354
**Radiological variables**						
Hunt and Hess grade IV and V	4.500 (1.690−11.983)	0.003	1.722 (0.332−8.938)	0.517	1.521 (0.315−7.344)	0.601
Fisher grade III and IV	1.736 (0.540−5.583)	0.355	11.702 (1.444−94.816)	0.021	8.140 (1.021−64.880)	0.048
Anterior circulation aneurysm	0.548 (0.129−2.323)	0.415	0.986 (0.129−7.513)	0.989	0.424 (0.045−3.979)	0.452
Hydrocephalus	1.660 (0.340−8.098)	0.531	3.457 (0.519−23.012)	0.200	3.177 (0.670−15.075)	0.146
Severe vasospasm	0.643 (0.201−2.058)	0.457	1.458 (0.325−6.550)	0.623	2.033 (0.526−7.847)	0.303
**Treatment methods**						
Coil embolization	0.621 (0.184−2.102)	0.444	1.227 (0.207−7.280)	0.822	1.360 (0.215−8.614)	0.744
**Haptoglobin**						
Relative α1 / albumin intensity	0.010 (0.000−0.522)	0.022	2.589 (0.116−57.989)	0.549	3.813 (0.312−46.644)	0.295

*, ** Six month-cognitive impairment was analyzed in 113 patients with Hp2-1, and long-term cognitive impairment was analyzed in 102 patients with Hp2-1. OR, odds ratio; CI, confidence interval; HR, hazards ratio.

## Discussion

The Hp phenotype was closely associated with long-term neurological outcomes following SAH and cognitive impairment in SAH survivors. Patients with Hp2-2 or Hp2-1 showed a significantly increased risk of long-term poor neurological outcomes compared with those carrying Hp1-1. In the case of Hp2-2, the possibility of cognitive impairment at 6 months and long term was significantly higher than in patients with Hp1-1, but this association was not statistically significant in patients with Hp2-1 compared with Hp1-1. The relative α1 intensity in patients with Hp2-1 was useful in predicting 6-month neurological outcomes.

Most of the studies of neurological outcomes by the Hp phenotype reported outcomes within 6 months of ictus. Outcomes after SAH are largely dependent on early brain injury and DCI (Eagles et al., 2019). Thus, the impact of the Hp phenotype on the outcome may be biased, especially in the early period after SAH, since the prognosis is affected more by the damage of the SAH than the Hp phenotype. In this study, we investigated long-term outcomes over a mean follow-up duration of 34.6 months. The Hp2-2 phenotype significantly increased the risk of poor neurological outcomes compared with Hp1-1. We further analyzed the association of Hp phenotypes with long-term outcomes based on dichotomized Fisher grades (I–II vs. III–IV). Amongst patients with lower Fisher grades (I and II), Hp2-2 (HR: 3.390, 95% CI: 0.441–26.040), and Hp2-1 (HR: 1.858, 95% CI: 0.217–15.875) did not show an increased risk of poor outcomes when compared with Hp1-1 as the reference. Conversely, the Hp phenotypes of patients with high Fisher grades (III and IV) were closely associated with long-term poor outcomes (Hp2-2, HR: 7.410, 95% CI: 1.752–31.341; and Hp2-1, HR: 5.244, 95% CI: 1.197–22.970).

Survivors of SAH were more vulnerable to dementia than those with ischemic stroke (Corraini et al., 2017). Based on an MMSE score of 27 or less, the rate of cognitive impairment is usually in the range of 10–20% (King et al., 2006; Eagles et al., 2019). Eagles et al. (2019) reported that DCI was a risk factor for cognitive impairment following SAH (Eagles et al., 2019). Buunk et al. (2019) also reported that complex attention and executive function were closely related to long-term return to work. The effect of Hp phenotype on cognitive function has yet to be established, although studies have investigated the relationship between cognition impairment and Hp phenotype in chronic diseases (Beeri et al., 2018). Beeri et al. (2018) reported that Hp1-1 increased the risk of poor cognitive function and decline compared with other Hp phenotypes in African American individuals with type 2 diabetes. However, our results showed that the presence of Hp2-2 increased the risk of 6-month and long-term cognitive impairment compared with Hp1-1. When the correlation between Hp phenotype and cognition was further analyzed according to age and gender, the association was more pronounced in younger patients and women.

**FIGURE 3:**
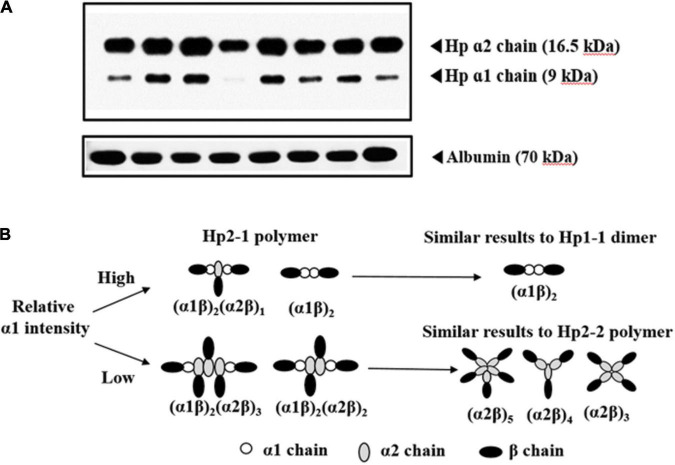
**(A)** Western blotting analysis of haptoglobin (Hp) 2-1 for determination of relative α1 intensity. Albumin level is used as the reference value **(B)**. Proposed mechanism of association with patient outcomes based on relative α1 intensity in patients with subarachnoid hemorrhage associated with Hp2-1 phenotype.

Compared with studies correlating Hp2-2 and Hp1-1, few studies have focused on Hp2-1. Although a lower incidence of DCI and vasospasm were observed in Hp2-1 compared with Hp1-1 (Ohnishi et al., 2013; Leclerc et al., 2015) a meta-analysis did not find a significant difference in DCI and vasospasm between these two Hp types (Gaastra et al., 2017). However, patients with SAH carrying Hp2-1 experienced a lower rate of DCI and vasospasm than those with Hp2-2 (Borsody et al., 2006; Murthy et al., 2016). These differences may be attributed to the structural characteristics of Hp2-1, which varies from dimers to polymers with different molecular weights (Kim et al., 2018). Previously, we reported that a relatively high α1 intensity was associated with a lower incidence of DCI and vasospasm compared with low α1 intensity in patients with Hp2-1 but not relative α2 intensities (Kim et al., 2018). Accordingly, we have analyzed outcomes in 126 patients with SAH expressing Hp2-1, focusing on relative α1 intensity. A 6-month poor outcome was associated with lower α1/albumin intensity and higher H-H grades (OR: 4.679, 95% CI: 1.735–12.620). We suspected that the predominant effect of Hp2-1 might be dependent on the relative α1 intensity mediated by the difference in Hp-Hb complex uptake by CD163. Hp2-1 with high α1 intensity tended to be associated with a lower molecular weight under denaturing conditions than those with lower α1 intensity (Kim et al., 2018). The lower molecular weights may contribute to diminished oxidative stress and better clearance of the Hp-Hb complex from the subarachnoid space compared with Hp2-1 with lower α1 intensities (Kim et al., 2018). In addition, anti-inflammatory effects were more pronounced in those carrying Hp1-1 than Hp2-2 (Asleh et al., 2003; Guetta et al., 2007). Therefore, we hypothesized that Hp2-1 with a higher relative α1 intensity acts similar to Hp1-1 and is correlated with better 6-month outcome (Figure 3). However, the relative α1 intensity was not associated with cognitive impairment following SAH.

In this study, we investigated the association between the Hp phenotype and outcomes in patients aged above or below 65 years and different gender since age at the time of disease and gender affect the incidence of dementia in SAH survivors (Corraini et al., 2017). Corraini et al. (2017) reported that stroke survivors aged ≥65 years (9.74%, 95% CI: 9.58–9.90) exhibited a significantly higher risk of 10-year dementia than those aged <64 years. Compared with ischemic stroke survivors, SAH survivors exhibited a higher relative risk of dementia. In particular, the risk of dementia was elevated within the first year after stroke onset in SAH survivors. In terms of gender influence on cognitive impairment, the risk of dementia is increased in women compared with men with advancing age (Fratiglioni et al., 2000). In addition, the effect of biological factors such as dementia-associated genetic variants on the occurrence of dementia may differ depending on gender. Brain autopsy studies have revealed more amyloid plaques and neurofibrillary tangles in women than in men who were carriers of the APOE e4 allele (Corder et al., 2004; Rocca et al., 2014). Our study also shows that age and gender affected SAH survival during the follow-up. Patients with SAH aged less than 65 years were more likely to experience cognitive impairment, suggesting the need for awareness of this in clinical practice.

The study has some limitations. First, we used a brief instrument, the MMSE, for the evaluation of cognitive outcomes. MMSE showed a higher sensitivity for assessing cognitive function in patients with SAH than telephone interview for cognitive status assessment (King et al., 2006) but may be inappropriate for the evaluation of frontal lobe function as it may be biased toward the functions associated with the left hemisphere in the brain including language, calculation, and verbal memory. Although complex neuropsychological tests such as the Seoul Neuropsychological Battery or Alzheimer’s Disease Assessment Scale-Cognitive Subscale can be used for the accurate evaluation of a patient’s cognitive function, they require specialized training, time, and space, as well as high cost. Thus, they are not widely used in actual clinical practice. Second, we defined cognitive impairment as a score of <27 on MMSE (Eagles et al., 2019). According to the original MMSE, a score <24 was an indicator of cognitive impairment (Folstein et al., 1975; King et al., 2006). However, even if patients manifest cognitive impairment in daily life, it is common in clinical practice to score 24 or higher in MMSE. Therefore, we believe that an MMSE cutoff of 27 reflects real-world conditions. Third, the association of DCI with Hp was not studied. DCI can be triggered by various causes such as microcirculatory dysfunction, glymphatic impairment, inflammation, and neuroelectric disruption as well as severe cerebral vasospasm. However, it is difficult to differentiate them in clinical practice. In addition, the criteria for diagnosing DCI differ across institutions, and the DCI treatment method depends on the clinician. Accordingly, severe vasospasm rather than DCI was used as a variable in the analysis.

## Conclusion

Patients diagnosed with SAH expressing the Hp2-2 phenotype showed a higher risk of poor neurological outcome and cognitive impairment than those expressing Hp1-1. The association between the Hp phenotype and cognitive impairment was more evident in women and subjects below 65 years of age. Within the subset of patients carrying Hp2-1, higher relative α1 intensities associated with a favorable neurological outcome at 6 months but not with cognition.

## Data Availability Statement

The original contributions presented in the study are included in the article/supplementary material, further inquiries can be directed to the corresponding author.

## Ethics Statement

The studies involving human participants were reviewed and approved by the Institutional Review Boards (IRB No: 2016-31, 2018-6, and 2019-06) of the Hallym University. The patients/participants provided their written informed consent to participate in this study.

## Author Contributions

JJ devised the original study design. SH, BK, TK, SL, JL, and DY collected the data. EH, JR, and JP performed the statistical analyses. YC and JJ interpreted the results. JJ and SH wrote the manuscript. BG and IG helped with data interpretation and made revisions. All authors contributed to the article and approved the submitted version.

## Conflict of Interest

The authors declare that the research was conducted in the absence of any commercial or financial relationships that could be construed as a potential conflict of interest.

## Publisher’s Note

All claims expressed in this article are solely those of the authors and do not necessarily represent those of their affiliated organizations, or those of the publisher, the editors and the reviewers. Any product that may be evaluated in this article, or claim that may be made by its manufacturer, is not guaranteed or endorsed by the publisher.
